# Pumpkin seed oil (*Cucurbita pepo*) versus tamsulosin for benign prostatic hyperplasia symptom relief: a single-blind randomized clinical trial

**DOI:** 10.1186/s12894-021-00910-8

**Published:** 2021-10-19

**Authors:** Nikan Zerafatjou, Mohammadali Amirzargar, Mahdi Biglarkhani, Farzaneh Shobeirian, Ghazal Zoghi

**Affiliations:** 1 Department of Urology, School of Medicine, Hamadan University of Medical Sciences, Hamadan, Iran; 2 Urology and Nephrology Research Center, Hamadan University of Medical Sciences, Hamadan, Iran; 3 Department of Persian Medicine, School of Medicine, Hamadan University of Medical Sciences, Hamadan, Iran; 4 Department of Radiology, School of Medicine, Guilan University of Medical Sciences, Rasht, Iran; 5 Endocrinology and Metabolism Research Center, Hormozgan University of Medical Sciences, Bandar Abbas, Iran

**Keywords:** Benign prostatic hyperplasia, Tamsulosin, Pumpkin seed oil

## Abstract

**Background:**

Benign prostatic hyperplasia (BPH) is very common in aging men. We aimed to compare the effects of tamsulosin and pumpkin (*Cucurbita pepo*) seed oil on BPH symptoms.

**Methods:**

This single-blind randomized clinical trial included patients with BPH aged ≥ 50 years referred to the Urology Clinic of Shahid Beheshti Hospital, Hamadan, Iran, from August 23, 2019 to February 19, 2020. Patients were randomized into two groups. One group received 0.4 mg tamsulosin every night at bedtime and the other received 360 mg pumpkin seed oil twice a day. Patients’ age, weight, height, and body mass index (BMI) were recorded. The International Prostate Symptom Score (IPSS) was filled out by the patients at baseline and then 1 month and 3 months after the initiation of treatment. The BPH-associated quality of life (QoL), serum prostate-specific antigen, prostate and postvoid residual volume, and maximum urine flow were also assessed at baseline and 3 months later. Drug side effects were also noted.

**Results:**

Of the 73 patients included in this study with a mean age of 63.59 ± 7.04 years, 34 were in the tamsulosin group and 39 in the pupkin seed oil group. Patients were comparable with respect to age, weight, height, BMI, and baseline principal variables in both groups. Also, there was no significant difference between groups in terms of principal variables at any time point. However, there was a significant decrease in IPSS and a significant improvement in QoL in both groups. Although the decrease in IPSS from baseline to 1 month and 3 months was significantly higher in the tamsulosin group compared to the pumpkin group (*P* = 0.048 and *P* = 0.020, respectively), the decrease in IPSS from 1 to 3 months was similar (*P* = 0.728). None of the patients in the pumpkin group experienced drug side effects, while dizziness (5.9%), headache (2.9%), retrograde ejaculation (2.9%), and erythema with pruritus occurred in the tamsulosin group.

**Conclusions:**

Pumpkin (*Cucurbita pepo*) seed oil relieved BPH symptoms with no side effects, but was not as effective as tamsulosin. Further studies are required to confirm the role of pumpkin seed oil as an option for the treatment of BPH symptoms.

*Trial registration* Iranian Registry of Clinical Trials, IRCT20120215009014N340. Registered 19.02.2020. Retrospectively registered, https://en.irct.ir/trial/45335.

**Supplementary Information:**

The online version contains supplementary material available at 10.1186/s12894-021-00910-8.

## Introduction

Benign prostatic hyperplasia (BPH) is a common age-dependent chronic disease that results from the progressive enlargement of the prostate gland due to the non-malignant proliferation of epithelial prostate cells and smooth muscle cells [1]. A recent systematic review and meta-analysis including data from 25 countries reported a lifetime prevalence of 26.2% for BPH [2]. Patients with BPH become symptomatic when the tissue overgrowth around the urethra constricts its opening leading to lower urinary tract symptoms (LUTS), including incomplete urination, frequency, urgency, nocturia, and decreased urine flow [3]. The prevalence of BPH-associated LUTS increases with age and it has been reported that approximately 80% of men experience BPH-associated LUTS by 70 years of age [4]. However, not every man with BPH symptoms seeks medical attention; most often, BPH patients only seek medical care when BPH-associated LUTS become bothersome or intolerable [1].

There is a variety of treatment strategies for BPH symptoms depending on symptom severity, together with patient discomfort and preference. These treatment strategies include lifestyle alterations, medical therapy, and surgical treatment [5]. Alpha-blockers such as tamsulosin are excellent first-line options of medical therapy [6]. Recently, there has been an increasing tendency towards the use of herbal medicines for different medical conditions. Pumpkin (*Cucurbita*) seeds are traditionally known around the world for their remedial effects on urinary tract complications, such as nocturia, urinary frequency, and stress urinary incontinence [7]. Pumpkins belong to the Cucurbitaceae family which includes various species such as *Cucurbita pepo*, *Cucurbita moschata*, and *Cucurbita maxima* [8]. *Cucurbita pepo* seed oil consists of high amounts of free fatty acids serving as a natural source of vitamins, proteins, trace elements, and polyunsaturated fatty acids, such as omega 3, 6, and 9 [9]. It is the phytosterol content of *Cucurbita pepo* seed oil that appears to interfere with the function of dihydrotestosterone produced by 5α-reductase which plays a major role in the process of BPH [10]. The phytosterol content varies among different *Cucurbita* species, including *Cucurbita pepo* and the previously mentioned *Cucurbita moschata* and *Cucurbita maxima* [11, 12]. However, only *Cucurbita pepo* is available in the Iranian market. Thus, in the current study we aimed to compare the effects of *Cucurbita pepo* with tamsulosin for the treatment of BPH symptoms.

## Methods

### Participants

This single-blind randomized clinical trial included patients aged ≥ 50 years with the clinical diagnosis of BPH by an expert urologist based on history, digital rectal examination (DRE), and paraclinical tests including serum prostate-specific antigen (PSA), who had been referred to the Urology Clinic of Shahid Beheshti Hospital, Hamadan, Iran, from August 23, 2019 to February 19, 2020. Exclusion criteria were PSA > 10 ng/ml, indications of surgical treatment (patients who were planned to receive surgical treatment for BPH due to absolute or relative indications, including renal insufficiency caused by benign prostatic obstruction, intractable urinary retention, recurrent cystitis, failure of medical therapy, bladder calculi, and persistent hematuria due to prostatic bleeding), previous surgical treatment of BPH, symptom exacerbation during the study period mandating surgical intervention, change in the diagnosis during the study period, any morbidities interfering with the course of treatment, having taken BPH-related medications within the past 6 months, and the development of drug side effects leading to its discontinuation. In patients with PSA > 4 ng/ml and < 10 ng/ml, in order to rule out prostatic cancer, DRE was done and free/total PSA ratio was measured. Also, in suspicious cases, prostatic biopsy was performed. The sample size was calculated as at least 35 patients in each group using a level of significance of 5%, power of 90%, and non-inferiority margin of 50%. The study was approved by the Institutional Review Board of Hamadan University of Medical Sciences (IR.UMSHA.REC.1398.429) and it complies with the statements of the Declaration of Helsinki. Written informed consent was obtained from all the participants. The study has also been retrospectively registered at the Iranian Registry of Clinical Trials (IRCT) under the registration number: IRCT20120215009014N340.

### Study design

**Fig. 1:**
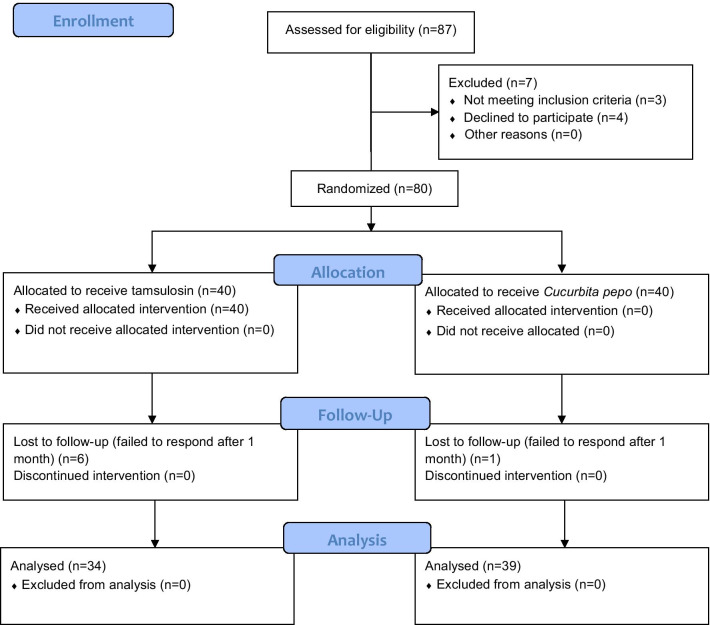
CONSORT 2010 Flow Diagram

Initially, 80 patients were eligible to enter the study. Patients were randomized into two equal groups using random generated numbers by the Random Allocation software. Seven patients were lost to follow-up. Details of patient enrollment are demonstrated in Fig. 1. Patients’ age, weight, and height were recorded. Body mass index (BMI) was calculated for each patient by dividing their weight (kg) by the square of their height (m). Patients in the tamsulosin group received 0.4 mg tamsulosin capsules (Farabi Pharmaceutical Co., Iran) every night at bedtime, while patients in the pumpkin group received 360 mg pumpkin (*Cucurbita pepo*) seed oil capsules (Tehran Darou Pharmaceutical Co., Iran) containing 1% phytosterol, twice a day. The reason for choosing this dose and type of pumpkin (*Cucurbita pepo*) was that it was the only form and dose available in the Iranian market. In previous studies, Vahlensieck et al. and Friedrich et al. used 1000 mg of pumpkin [13, 14]; however, with the available dose per capsule in our study, taking 3 capsules (1080 mg) could have reduced patient compliance. Thus, patients in the pumpkin group received 2 capsules a day (720 mg). Patients in both groups continued taking their medications till the last round of evaluations at 3 months. Patients were asked to fill out the International Prostate Symptom Score (IPSS) for the assessment of BPH symptom severity, before treatment and then 1 and 3 months later. The BPH-associated quality of life (QoL) scale was also used once before treatment and then at 3 months. The QoL scale evaluates how the patient would feel if he were to spend the rest of his life with his current urinary condition. It is scored from 0 to 6 with 0 indicating the highest and 6 the lowest QoL. Before treatment and at 3 months, all patients underwent ultrasonography for the measurement of postvoid residual (PVR) and prostate volume. Uroflowmetry was also performed for all the participants at the same time points and serum PSA was measured as well. Drug side effects, including dizziness, headache, retrograde ejaculation, and erythema with pruritus during the study period were noted. The laboratory personnel and the individuals in charge of ultrasonography and uroflowmetry were blinded to the grouping of the patients.

### Data analysis

We used the Statistical Package for the Social Sciences (SPSS) software (version 25.0, Armonk, NY: IBM Corp.) for data analysis. Mean, standard deviation, frequency, and percentages were used to describe the variables. Based on the results of Kolmogorov–Smirnov normality test, independent *t* test and Mann–Whitney test were used to compare quantitative variables between groups. Friedman test was used to assess the significance of changes in qualitative variables across different time points. Pairwise comparisons were made using paired t-test and Wilcoxon test. *P* values ≤ 0.05 were regarded as statistically significant.

## Results

Of the 73 patients included in this study with a mean age of 63.59 ± 7.04 years, 39 (53.4%) were in the pumpkin group and 34 (46.6%) in the tamsulosin group. Patients’ general characteristics are shown in Table 1. Participants in both groups were comparable regarding age, weight, height, and BMI.

**Table 1:** General characteristics of the study population

Variables	Total (n = 73)	Tamsulosin (n = 34)	Pumpkin (n = 39)	*P* value*
Age (years) mean ± SD	63.59 ± 7.04	62.71 ± 6.63	64.36 ± 7.38	0.320
Height (cm) mean ± SD	169.49 ± 6.74	169.12 ± 6.49	169.82 ± 7.01	0.916†
Weight (kg) mean ± SD	73.04 ± 12.21	71.27 ± 12.11	74.54 ± 12.25	0.261
BMI (kg/m^2^) mean ± SD	25.38 ± 3.64	24.89 ± 3.68	25.78 ± 3.61	0.309

n, number; SD, standard deviation; BMI, body mass index

^*^Analyzed by independent t-test

^†^Analyzed by Mann–Whitney test

There was no significant difference between groups in terms of baseline IPSS, IPSS at one month, and IPSS at three months (Table 2). However, there was a significant change in IPSS from baseline to the end of three months in both groups (*P* < 0.001). The reduction in IPSS from baseline to 1 month and 3 months was significantly higher in the tamsulosin group compared to the pumpkin group (− 3.30 ± 3.15 vs. − 1.74 ± 3.02, *P* = 0.048 and − 5.33 ± 3.64 vs. − 3.19 ± 3.63, *P* = 0.020, respectively). Pairwise comparisons (Additional file 1: Table S1) also showed significant decrease in IPSS between any two time points in both groups. Moreover, although the QoL scores were comparable between groups at baseline and at 3 months, these scores significantly decreased between these time points in both groups (Table 2). Serum PSA was also similar in both groups at baseline and at 3 months. There was a slight increase in serum PSA between these time points in both groups; nevertheless, this was not statistically significant. Prostate volume was comparable between groups at baseline and at 3 months with no significant increase in any of the groups. The same was true for PVR and maximum urine flow.

**Table 2:** Comparison of IPSS, QoL, serum PSA, maximum urine flow, PVR, and prostate volume between groups at different time points

Variables	Tamsulosin (n = 34)	Pumpkin (n = 39)	P-value*
	Mean ± SD (95% CI)	Mean ± SD (95% CI)	
Baseline IPSS	10.58 ± 5.70 (8.55–12.60)	11.08 ± 5.71 (9.23–12.93)	0.773
IPSS at 1 month	7.35 ± 4.53 (5.77–8.93)	9.33 ± 5.57 (7.53–11.14)	0.174
IPSS at 3 months	5.65 ± 4.25 (4.09–7.20)	7.46 ± 5.84 (5.51–9.41)	0.231
P-value	< 0.001†	< 0.001†	
IPSS change from baseline to 1 month	− 3.30 ± 3.15 (− 4.42–− 2.19)	− 1.74 ± 3.02 (− 2.72–− 0.77)	0.048
IPSS change from baseline to 3 months	− 5.33 ± 3.64 (− 6.69–− 3.97)	− 3.19 ± 3.63 (− 4.40–− 1.98)	0.020
IPSS change from 1 to 3 months	− 1.84 ± 2.59 (− 2.79–− 0.89)	− 1.46 ± 3.81 (− 2.73–− 0.19)	0.728
Baseline QoL score	2.35 ± 1.41 (1.86–2.85)	2.41 ± 1.33 (1.98–2.84)	0.767
QoL score at 3 months	1.38 ± 0.99 (1.04–1.73)	1.67 ± 1.06 (1.32–2.01)	0.148
P-value	< 0.001‡	0.001‡	
QoL change	− 0.97 ± 1.03 (− 1.33–− 0.61)	− 0.74 ± 1.16 (− 1.12–− 0.37)	0.465
Baseline serum PSA (ng/ml)	2.39 ± 1.57 (1.80–2.99)	2.91 ± 2.54 (2.05–3.77)	0.678
Serum PSA at 3 months (ng/ml)	2.67 ± 2.16 (1.81–3.52)	3.05 ± 2.88 (1.83–4.26)	0.706
P-value	0.194‡	0.903‡	
Serum PSA change (ng/ml)	0.48 ± 1.41 (− 0.11–1.07)	0.13 ± 1.36 (− 0.47–0.73)	0.396¶
Baseline prostate volume (ml)	50.93 ± 22.74 (42.87–58.99)	53.53 ± 23.53 (45.79–61.26)	0.511
Prostate volume at 3 months (ml)	55.00 ± 21.22 (46.21–63.75)	58.32 ± 23.78 (46.85–69.77)	0.629¶
P-value	0.569‡	0.180§	
Prostate volume change (ml)	0.38 ± 11.43 (− 4.44–5.20)	3.22 ± 9.77 (− 1.63–8.08)	0.402¶
Baseline PVR (ml) mean ± SD	67.56 ± 71.32 (38.12–97.00)	67.03 ± 53.67 (46.62–87.45)	0.385
PVR at 3 months (ml)	56.43 ± 81.63 (19.27–93.59)	51.61 ± 33.54 (34.93–68.29)	0.080
P-value	0.723‡	0.087‡	
PVR change (ml)	− 9.56 ± 94.93 (− 56.76–37.65)	− 7.67 ± 21.98 (− 19.84–4.50)	0.563
Baseline maximum urine flow (ml/sec)	12.97 ± 11.41 (8.45–17.48)	9.44 ± 4.92 (7.69–11.18)	0.172
Maximum urine flow at 3 months (ml/sec)	12.27 ± 5.62 (9.83–14.69)	11.18 ± 6.09 (8.61–13.75)	0.344
P-value	0.698‡	0.091‡	
Maximum urine flow change (ml/sec)	0.11 ± 3.09 (− 1.42–1.64)	1.82 ± 4.39 (− 0.12–3.77)	0.328

SD, standard deviation; CI, confidence interval; IPSS: International Prostate Symptom Score; QoL, quality of life; PSA, prostate-specific antigen; PVR, postvoid residual

*Analyzed by Mann–Whitney test

^†^Analyzed by Friedman test

^‡^Analyzed by Wilcoxon test

§ Analyzed by paired t-test

^¶^Analyzed by independent *t* test

Furthermore, none of the patients in the pumpkin group experienced drug side effects, while in the tamsulosin group, dizziness occurred in 2 patients (5.9%), headache in 1 (2.9%), retrograde ejaculation in 1 (2.9%), and erythema with pruritus in 1 (2.9%).

## Discussion

In the current study, we found that both tamsulosin and pumpkin (*Cucurbita pepo*) seed oil significantly reduced BPH symptoms assessed by IPSS. However, the decrease in IPSS from baseline to 1 month and 3 months was significantly higher in the tamsulosin group compared to the pumpkin group, while the decrease from 1 to 3 months was similar. Moreover, no patients in the pumpkin group experienced drug side effects, while dizziness (5.9%), headache (2.9%), retrograde ejaculation (2.9%), and erythema with pruritus occurred in the tamsulosin group.

With a global prevalence of 20–62% in men over 50 years, BPH is considered a very common condition [15]. BPH causes LUTS which markedly affect the quality of life in many patients [16, 17]. Multiple strategies have been proposed and used for the relief of BPH-related symptoms. Alpha-blockers such as tamsulosin are regarded as the first-line options. By selectively blocking α_1_A-adrenergic receptors leading to the relaxation of the prostate smooth muscles, tamsulosin is reported to improve dysuria and other BPH symptoms [18]. Although tamsulosin is generally preferred due to its lower side effects compared to other alpha-blockers, it can still cause some complications and unwanted reactions, including dizziness, headache, and retrograde ejaculation [19]. We also observed some of these side effects in patients taking tamsulosin.

The effects of tamsulosin and pumpkin seed oil on BPH symptoms have separately been investigated in multiple studies, yet none have compared them with regard to BPH symptom relief. In vitro and in vivo experiments have shown promising results for pumpkin seeds. In their study on Sprague–Dawley rats, Gossell-Williams et al. observed a reduction of prostate size with pumpkin seed oil [20]. This effect has been confirmed by other in vitro and animal studies [21–23]. In line with our findings, Vahlensieck et al. demonstrated a clinically relevant reduction of IPSS compared to placebo after 12 months of taking pumpkin seed extract [13]. They used 500 mg *Cucurbita pepo* seed oil extract capsules twice a day. Nevertheless, the period of treatment was much longer in their study reflecting the long-term effects of pumpkin seeds compared to the 3-month period of treatment in our study. In a study on 100 patients by Shirvan et al., pumpkin seed oil (360 mg *Cucurbita pepo* twice a day) was compared with prazosin for the treatment of BPH symptoms [24]. They found pumpkin seed oil to be safe and effective but not as effective as prazosin. Their findings were consistent with ours. We also found a significant reduction in IPSS with both tamsulosin and pumpkin seed oil from baseline 3 months; nonetheless, the reduction in IPSS from baseline to the end of 1 month and 3 months was significantly higher with tamsulosin. Moreover, they further evaluated patients after 6 months of ttreatment, while we only assessed the results after 3 months. The results of a large study by Friederich et al. on 2245 BPH patients taking pumpkin seed extract (1–2 500 mg *Cucurbita pepo* capsules) for 3 months have also confirmed the effectiveness of this herbal medications showing a 41.4% decrease in IPSS and 46.1% improvement in their QoL [14]. Dihydrotestosterone, converted from testosterone by 5α-reductase, is responsible for the overgrowth of the prostate gland which is characteristic of BPH. [25]. Targeting this pathway has been the mainstay of medical treatment in BPH. Pumpkin seeds appear to affect BPH through the same pathway [10]. Anti-inflammatory properties of pumpkin seeds have been proposed as another mechanism for the effectiveness of pumpkin seed oil in BPH [26]. BPH is associated with the inflammation of the prostate gland and overexpression of cytokines, leukotriene, inducible nitric oxide synthase, NF-κB, and cyclooxygenase-2 is linked with prostatitis [27]. Another proposed mechanism is the diuretic effects of pumpkin seed due to its fatty acids content [28].

Of note, IPSS is a subjective tool to assess the improvement of BPH symptoms. An objective method to evaluate BPH improvement is maximum urine flow and we found no significant change in this variable in any of the groups. One reason for this can be that we had included patients with > 15 ml/s maximum urine flow at baseline only because they complained of BPH symptoms and this could have interfered with the results.

Also, we did not observe significant reductions in PSA and prostate volume with pumpkin seed oil. The results of the study by Hong et al. were similar, as they found no significant reduction in PSA and prostate volume after 3 months in patients receiving 320 mg/day pumpkin seed oil. They did not specify the *Cucurbita* species used in their study (29). As the effect of pumpkin seed oil has been attributed to its phytosterol content, higher doses of pumpkin seed oil potentially include higher amounts of phytosterol leading to increased effects. Nevertheless, patients’ adherence to medications would be of concern with higher doses.

The strength of the current study was the homogeneity of patients taking tamsulosin or pumpkin seed oil regarding the potential effectors, as well as baseline characteristics, including symptom severity, QoL, serum PSA, maximum urine flow, and prostate and PVR volumes. This study was not without limitations. First, it has been conducted on a limited number of patients. The study did not reach the minimal sample size of 35; 6 patients were lost to follow-up in the tamsulosin group and 34 patients remained in this group. Further studies with a larger sample size are required to confirm our findings. Second, we only evaluated the efficacy of pumpkin seeds for a 3-month period of treatment and its long-term effects need to be determined in future studies. Also, patients’ adherence to treatment, which we failed to assess, is another factor that could have influenced the results. Moreover, we included patients with IPSS < 8 who were mildly symptomatic. Although the initial design of the study was to only include patients with moderate to severe BPH symptoms, we also included mildly symptomatic patients due to the persistent complaint of these patients of BPH symptoms causing them discomfort, as well as the limited recruitment time.

## Conclusions

Both pumpkin seed oil and tamsulosin significantly reduced BPH symptoms; however, due the higher reduction in IPSS scores from baseline to 1 month and 3 months, tamsulosin was more effective. The advantage of pumpkin seed was its lower side effects of pumpkin seeds. Further studies are required to confirm the role of pumpkin seed oil as an option for the treatment of BPH symptoms.

## Supplementary Information

**Additional file 1.** Pairwise comparisons of IPSS scores at different time points in each group.

## Data Availability

The datasets used and/or analyzed during the current study are available from the corresponding author on reasonable request.

## Abbreviations

BMI: Body mass index
BPH: Benign prostatic hyperplasia
DRE: Digital rectal examination
IPSS: International Prostate Symptom Score
IRCT: Iranian Registry of Clinical Trials
LUTS: Lower urinary tract symptoms
PSA: Prostate-specific antigen
PVR: Postvoid residual
QoL: Quality of life
