# Supplementary material for: Pumpkin seed oil (Cucurbita pepo) versus tamsulosin for benign prostatic hyperplasia symptom relief: a single-blind randomized clinical trial
Source: BMC Urol. 2021 Oct 19;21:147. doi: 10.1186/s12894-021-00910-8 (PMC8527717; doi:10.1186/s12894-021-00910-8)
Supplement: Supplementary file 1 — Additional file 1. Pairwise comparisons of IPSS scores at different time points in each group. [file 12894_2021_910_MOESM1_ESM.docx]

Supplementary Table 1: Pairwise comparisons of IPSS scores at different time points in each group

| Tamsulosin | P-value* |
| --- | --- |
| IPSS at baseline with 1 month | <0.001 |
| IPSS at baseline with 3 months | <0.001 |
| IPSS at 1 month with 3 months | 0.001 |
| Pumpkin |  |
| IPSS at baseline with 1 month | <0.001 |
| IPSS at baseline with 3 months | <0.001 |
| IPSS at 1 month with 3 months | 0.018 |

Abbreviations: IPSS: International Prostate Symptom Score.

*Analyzed by Wilcoxon test.
